# Recombinant Hamster Oviductin Is Biologically Active and Exerts Positive Effects on Sperm Functions and Sperm-Oocyte Binding

**DOI:** 10.1371/journal.pone.0123003

**Published:** 2015-04-07

**Authors:** Xiaojing Yang, Yuewen Zhao, Xiaolong Yang, Frederick W. K. Kan

**Affiliations:** 1 Department of Biomedical and Molecular Sciences, Faculty of Health Sciences, Queen’s University, Kingston, Ontario, Canada; 2 Department of Pathology and Molecular Medicine, Faculty of Health Sciences, Queen’s University, Kingston, Ontario, Canada; Cornell University College of Veterinary Medicine, UNITED STATES

## Abstract

Studies carried out in several mammalian species suggest that oviductin, also known as oviduct-specific glycoprotein or OVGP1, plays a key role in sperm capacitation, fertilization, and development of early embryos. In the present study, we used recombinant DNA technology to produce, for the first time, recombinant hamster OVGP1 (rHamOVGP1) in human embryonic kidney 293 (HEK293) cells. rHamOVGP1 secreted in the culture medium was purified by affinity chromatography. The resulting protein migrated as a poly-dispersed band of 160-350 kDa on SDS-PAGE corresponding to the molecular mass of the native HamOVGP1. Subsequent mass spectrometric analysis of the purified rHamOVGP1 confirmed its identity as HamOVGP1. Immunocytochemistry demonstrated binding of rHamOVGP1 to the mid-piece and head of hamster sperm and to the zona pellucida (ZP) of ovarian oocytes. *In vitro* functional experiments showed that addition of rHamOVGP1 in the capacitation medium further enhanced tyrosine phosphorylation of two sperm proteins of approximately 75 kDa and 83 kDa in a time-dependent manner. After 3 hours of incubation in the presence of rHamOVGP1, a significant increase in acrosome reaction was measured. Pretreatment of either sperm or oocyte with 20 μg/ml of rHamOVGP1 prior to sperm-egg binding assay significantly increased the number of sperm bound to the ZP. Addition of rHamOVGP1 in the medium during sperm-egg binding with either oocyte or sperm pretreated with rHamOVGP1 also saw an increase in the number of sperm bound to ZP. In all experimental conditions, the effect of rHamOVGP1 on sperm-oocyte binding was negated by the addition of monoclonal anti-HamOVGP1 antibody. The successful production and purification of a biologically active rHamOVGP1 will allow further exploration of the function of this glycoprotein in reproductive function.

## Introduction

The mammalian oviduct is a strategic site in the female reproductive tract where it provides a luminal microenvironment for gamete transport and maturation, sperm capacitation, and fertilization as well as early embryo development by secreting an oviductal fluid consisted of a combination of plasma exudates and secretory components from oviductal epithelial cells [1–3]. A major component of the secretory products of the oviduct is a high-molecular-weight, oviduct-specific and estrogen-dependent glycoprotein known as oviductin or oviduct-specific glycoprotein (OVGP1). OVGP1 has been identified in a variety of species, including the mouse [4, 5], hamster [6–9], rabbit [10], cow [11, 12], pig [13, 14], baboon [15, 16], rhesus monkey [17], goat [18], and human [19, 20]. OVGP1 belongs to the glycosyl hydrolase family 18 that lacks chitinase enzyme activity [21]. OVGP1 cDNAs have been cloned from several mammalian species and notable conservation has been found within the *N*-terminal amino acid sequences of different mammalian OVGP1 cDNAs whereas sequence divergence and low identity between species exist within the C-terminal regions [19, 22, 23]. Previous studies have shown that, upon its secretion into the lumen of the oviduct, mammalian OVGP1 becomes intimately associated with the zona pellucida (ZP) of postovulatory oocytes in oviductal transit [24], but is absent in ovarian oocytes [6, 25, 26]. Results from *in vitro* functional studies indicated that OVGP1 has positive effects on sperm capacitation, sperm-ovum binding, sperm penetration of the ovum, polyspermy prevention, and early embryo development [22, 23]. For examples, bovine OVGP1 has been shown to enhance sperm capacitation and increase *in vitro* fertilization rates [27]. *In vitro*, Hamster OVGP1 (HamOVGP1) has been shown to increase the sperm penetration rate three-fold as compared to the penetration rate in the absence of HamOVGP1 [28]. The presence of partially purified human OVGP1 during sperm-hemizona binding was found to enhance the binding of sperm to the outer ZP by a factor of 3.7, and the effect can be blocked by preincubation of human OVGP1 with antibody against human OVGP1 [29]. The presence of goat OVGP1 in the culture medium was found to enhance embryo cleavage rate and blastocyst formation and prevent polyspermy [18]. The secretion of equine oviductal epithelial cells was shown to increase the rate of *in vitro* fertilization [30]. Incubation of porcine *in vitro*-matured oocytes in oviductal fluid rendered the ZP resistant to proteolytic digestion and increased the incidence of monospermy [31]. Recent studies in our laboratory have demonstrated that estrus stage-specific HamOVGP1 enhances tyrosine phosphorylation of a sub-set of sperm proteins during *in vitro* capacitation [32]. These accumulative findings all point to several important roles played by OVGP1 during the early events of mammalian reproduction. Despite the identification of OVGP1 in various mammalian species, further exploration of its roles in mammalian reproduction and elucidation of the mechanism underlying its functions are hampered by the limited availability of sufficient amounts of purified OVGP1. To circumvent the problem of obtaining adequate amounts of native OVGP1 for future studies, the production of recombinant OVGP1 can be envisaged as an alternative.

In the present study, we successfully employed recombinant DNA technology to produce recombinant HamOVGP1 (rHamOVGP1) in human embryonic kidney (HEK293) cells. We further purified rHamOVGP1 from culture medium by lectin-affinity purification. To find out if rHamOVGP1 is biologically active, we examined the effect of rHamOVGP1 on capacitation by determining whether rHamOVGP1 can enhance tyrosine phosphorylation of sperm proteins which is a hallmark of capacitation. The effect of rHamOVGP1 on acrosome reaction of hamster sperm was also investigated. An additional aim of our study was to determine whether rHamOVGP1 could bind to the ZP of hamster ovarian oocytes and influence sperm-egg binding. Information gained from the present study shows that rHamOVGP1 is biologically active and that the large scale production of rHamOVGP1 may prove useful for further understanding of its role in fertilization and early embryo development as well as for elucidating the mechanism that regulates its functions.

## Materials and Methods

### Animals and reagents

Golden hamsters (*Mesocricetus auratus*) were purchased from Charles River (St. Constant, Quebec, Canada). Male and female hamsters of 7 to 9 weeks of age were housed in a temperature-controlled room with exposure to light 12 hours/day (6:00 a.m.-6:00 p.m.). All experiments carried out with the hamsters were approved by The University Animal Care Committee of Queen’s University in accordance with the guidelines stipulated by the Canadian Council on Animal Care. All reagents and chemicals were of molecular biology grade and were purchased from Fisher Scientific Co., Sigma-Aldrich, Invitrogen, or New England BioLabs Inc., unless otherwise specified. The monoclonal antibody (IgG_1_,^k^) against HamOVGP1 used in the present study was a gift from Dr. Gilles Bleau of the University of Montreal. The monoclonal antibody recognizes an antigen, an oviduct-specific glycoprotein the molecular weight of which obtained by SDS-PAGE under reducing conditions is between 160–350 kDa [33]. This glycoprotein contains a high proportion of sugar residues (85%) which account for the antigenic determinants recognized by the monoclonal antibody [8]. Tissue-specificity of the monoclonal antibody has been previously documented [33].

### Plasmid construction

HamOVGP1-pGEM-T was a gift from Dr. Gilles Bleau of the University of Montreal. HamOVGP1 cDNA possessing a partial open reading frame of HamOVGP1 with nucleotides 15–1766 of the sequence [34] was first amplified by PCR using HamOVGP1-pGEM-T plasmid as a template, digested by Pme I, and subsequently cloned into the Pme I site of WPI lentiviral vector, which bicistonically expresses green fluorescent protein (GFP). The HamOVG1-WPI plasmid was confirmed by DNA sequencing (ACGT CORP., Toronto, ON, Canada). The following primers were used for PCR: forward primer (5′-GA TAC CAT GTTTAAAC ATG CAT CAT CAC CAT CAC CAC GCT GAG ATG GGG AGG CTG CTG CTG-3′) and reverse primer (5’-GA AGT CAT GTTTAAAC CAC TGT GGC TGT GAT CTG TC-3’).

### Cell culture, transfection, and establishing the rHamOVGP1 stable cell line

The human embryonic kidney 293 cells (HEK293T; ATCC) were cultured at 37°C and 5% CO_2_ in Dulbecco’s Modified Eagle Medium (DMEM) containing 10% fetal bovine serum (FBS) and 1% Penicillin and Streptomycin. Lentiviruses were produced by Lipofectamine 2000-mediated transfection of HEK 293 as previously described by Chow *et al*. [35]. One day prior to transfection, 1.5 x 10^7^ HEK 293 cells were seeded in a 15 cm Petri dish in 25 ml of DMEM with 10% FBS. The next day, the cells were co-transfected with the lentiviral transfer plasmid pWPI plus packaging plasmid psPAX and envelop plasmid pMD2G at a ratio of 4:3:1 (w/w/w) according to manufacturer’s protocol. At 24 hours (h) post-transfection, the medium was replaced with 15 ml of warm OPTI-MEM I medium (serum free) containing 10 mM sodium butyrate. The medium was harvested at 48 h post-transfection and centrifuged at 500 xg for 10 minutes (min). The supernatant was carefully removed, filtered (0.45 μm membrane) to remove cellular debris, centrifuged at 1000 xg for 30 min and stored at -80°C as lentivirus stock solution for subsequent infection.

For the lentivirus infection of HEK 293 cells, a total of 1 x 10^5^ cells per well were seeded in a 6-well plate the night prior to infection and the cells were 40–50% confluent on the day of infection. The medium was removed and replaced with OPTI-MEM with polybrene (8 μg/ml) containing lentivirus stock solution. The cells were incubated in 5% CO_2_ at 37°C. At 48 h post-infection, 3 ml of growth medium (DMEM with 10%FBS, 1% Penicillin and Streptomycin) per well was added. The infected cells were sub-cultured into 10 cm dishes after they were confluent and the green fluorescent protein (GFP)-positive cells were monitored by fluorescence microscopy. About 2 weeks after the passage, GFP-positive cell clones were apparent. Using cloning cylinders individual GFP-positive clones were transferred to 96-well plates and grown in growth medium until confluence. The cells were then passaged into 24-well plates and continually grown in growth medium for 1–2 additional passages with daily monitoring of GFP-positive cell clones. The clones were screened for expression levels of rHamOVGP1 by immunoblot analysis of culture supernatants using monoclonal antibody against hamster OVGP1 [33]. HEK293 cells derived from clones stably expressing high levels of rHamOVGP1 were selected and maintained in the medium and GFP expression was monitored on a daily basis.

For large scale production of rHamOVGP1, the stable rHamOVGP1-expressing HEK293 cells were cultured in 15 cm dishes in CD293 serum-free medium supplemented with 4 mM L-glutamine, penicillin (50 U/ml), and streptomycin (50 μg/ml) at 37°C and 5% CO_2_. After 7–10 days, serum-free culture medium containing the recombinant glycoprotein was collected and centrifuged at 1000 xg for 5 min at 4°C. The culture supernatant was collected from each dish and stored at -70°C for further purification. Secretion of rHamOVGP1 was monitored weekly by Western blot analysis of culture supernatants.

### Purification of secreted rHamOVGP1 from serum-free cell culture medium by affinity chromatography

Purification of rHamOVGP1 was performed using lectin-affinity chromatography as previously described [36]. Briefly, the culture supernatant was dialyzed overnight in the cold room against a buffer containing 150 mM NaCl, 50 mM Tris-HCL (pH 8.0) and 0.02% NaN_3_. The buffer-exchanged solution was then concentrated by ultra-filtration using Amicon Ultra-15 centrifugal filter with 50 kDa cut-off membrane (EMD Millipore). The concentrated samples were loaded on *Helix pomatia agglutinin* (HPA)-agarose (5 ml; 1.5 mg/ml of lectin) column pre-equilibrated with the same buffer. Samples were incubated with HPA-agarose for 1 h at 4°C and the column was then washed extensively with 10 bed volume of buffer consisted of 300 mM NaCl, 50 mM Tris-HCl (pH 8.0), and 0.02% NaN_3._ rHamOVGP1 was eluted from HPA-agarose column with 3 bed volume of buffer consisted of 150 mM NaCl, 200 mM N-Acetyl-D-galactosamine (α-D-GalNAc, MP Biomedicals), 200 mM glycine-HCl (pH 2.5), and 0.02% NaN_3._ The flow rate of the column was controlled at 10 ml/h. The eluates were neutralized, desalted and concentrated by ultra-filtration using Amicon Ultra-15 centifugal filter with 50 kDa cut-off membrane.

### Identification of rHamOVGP1 by immunoblot and mass spectrometric analysis

The protein samples were size-fractionated by SDS-PAGE on a 6.0% gel. The proteins were visualized with silver staining. For Western blot analysis, the protein samples were mixed with reducing SDS sample buffer (2% SDS, 10% glycerol, 63 mM Tris HCl (pH 6.8), 0.1% β-mercaptoethanol, 0.0025% bromophenol blue), boiled for 5 min, separated on the gel, and electrophoretically transferred to polyvinylidene fluoride (PVDF) membrane. After blocking for 1 h with blocking buffer (5% nonfat milk and 0.1% Tween 20 in Tris-buffered saline (TBS) containing 50 mM Tris and 150 mM NaCl, pH 7.5), blots were probed sequentially with mouse monoclonal antibody against HamOVGP1 at a final concentration of 1 μg/ml, and then with peroxidase-labeled goat anti-mouse IgG at a final concentration of 0.02 μg/ml. Labeling was visualized by enhance chemiluminescence (ECL, PerkinElmer).

Purified rHamOVGP1 was further identified by mass spectrometry (MS). Briefly, the samples were run on SDS-PAGE gels. After brief staining of the gels, protein bands were cut from the gel and digested using the Micromass MassPREP Robotic Protein Handling System (PerkinElmer). The trypsin-digested sample was subjected to analysis using the SCIEX Voyager DE Pro Matrix-Assisted Laser-Desorption (MALDI) mass spectrometer at the Protein Function Discovery Facility of Queen’s University, Ontario, Canada. Data from peptide mass fingerprinting were acquired over the mass range m/z 700 to 4000. MS data were processed using Applied Biosystems Data Explorer version 5.1 and submitted to the Genebio Aldente search engine for comparison against the Swiss-Prot database.

### Preparation of sperm from the caudal epididymis

Motile hamster sperm were isolated and prepared following the procedure described by Bavister [37] with some modifications. Briefly, male hamsters at 7 to 9 weeks of age were anesthetized with an intraperitoneal injection of 150 μl sodium pentobarbital (McGill University, Montreal, Quebec, Canada) and then sacrificed by cervical dislocation. The epididymides were excised and placed in a 6 mm plastic petri-dish containing mineral oil extracted with Tyrode-lactate-HEPES-polyvinyl alcohol (TL-HEPES-PVA) buffer. The viscous cauda epididymal contents (CECs) were released into the oil by repeatedly puncturing the cauda epididymis with a 23-gauge syringe needle. CECs were then transferred into a polypropylene tube containing 5 ml of TL-HEPES-PVA medium and resuspended gently, and incubated for 5 min at 37°C. During this period, sperm were allowed to swim-up. The top layer (3 ml) of the suspension was withdrawn with a pipette and centrifuged at 500 xg for 5 min. After centrifugation, the supernatant was immediately removed and discarded. The sperm pellet was resuspended in Tyrode’s-Albumin-Lactate-Pyruvate (TALP) medium. TALP is a modified Tyrode’s medium containing NaCl (114 mM), KCl (3.16 mM), CaCl_2_ (2 mM), MgCl_2_ (0.5 mM), NaHCO_2_ (25 mM), NaH_2_PO_4_ (0.35 mM), sodium lactate (10 mM), glucose (5 mM), and phenol red (0.001g/100 ml, pH 7.5). TALP medium was always supplemented with PVA (0.1g/100 ml) and freshly made just before use or stored at 4°C and used within a month. Immediately prior to use, sodium pyruvate (0.1 mM) and bovine serum albumin (BSA) (3 mg/ml) were added and the medium was allowed to equilibrate at 37°C in 5% CO_2_ for 1 h. The motility stimulators PHE (100 x stock solution) namely D-penicillamine (2 mM), hypotaurine (10 mM) and epinephrine (100 μM) were added at 1/100 dilution in the medium equilibration. The vast majority of sperm (averaged 70–80%) were found to be motile when aliquots of the samples were viewed on the light microscope. The sperm count was determined using motile sperm obtained with the swim-up method.

### Tyrosine phosphorylation of sperm proteins during in vitro capacitation

A dose-dependent experiment was first carried out to determine the optimal concentration of rHamOVGP1 to be used throughout the investigation. Aliquots of the sperm suspension (2 x 10^6^ cells) were capacitated *in vitro* by incubating sperm in TALP-PVA medium containing PHE in the presence of different concentrations of rHamOVGP1 (0, 10, 20, 40, 60 μg/ml) for 4 h at 37°C in 5% CO_2_ with 100% humidity. The effect of different concentrations of rHamOVGP1 on enhancement of tyrosine phosphorylation of sperm proteins was assessed by Western blot analysis following the procedures described below. Results showed that rHamOVGP1 at a concentration of 20 μg/ml yielded the optimal results (see Results section). Accordingly, rHamOVGP1 at a concentration of 20 μg/ml was used throughout the rest of the investigation. To determine the time-dependent effects of rHamOVGP1 on tyrosine phosphorylation of sperm proteins, sperm were incubated in the capacitating medium in the presence of 20 μg/ml of rHamOVGP1 for 0–6 h. Aliquots of the sperm suspension were taken at different time points and processed for SDS-PAGE or indirect immunofluorescence described below. All experiments were repeated three to four times using different male hamsters.

### SDS-PAGE and immunoblotting of tyrosine phosphorylated sperm proteins

At the end of capacitation, sperm suspensions were collected and washed by centrifugation twice at 800 ×g for 5 min in D-PBS (Dulbecco’s phosphate-buffered saline, pH 7.4, containing 137 mM NaCl, 2.7 mM KCl, 8.1 mM Na_2_HPO_4_, 1.47 mM KH_2_PO_4_, 0.9 mM CaCl_2_, 0.5 mM MgCl_2_) with 1 mM sodium orthovanadate as a tyrosine phosphatase inhibitor. Sperm pellets were mixed with reducing SDS sample buffer (80 mM Tris HCl, pH6.8, 10% glycerol, 2% SDS, 5% β-mercaptoethanol, 0.02% bromophenol blue) and boiled for 5 min. In addition, demembranation of sperm was performed according to the procedure previously described [38]. The membrane was extracted twice for 15 min with shaking at 4°C in DT (2% Triton X-100, 5 mM DTT, and 50 mM Tris-HCl, pH9.0). Each extraction was followed by a centrifugation at 1,000 xg for 10 min. After the last extraction, the demembranated sperm pellet was washed twice in 50 mM Tris-HCl (pH9.0) and used for immunoblot analysis. Samples were analyzed on 7.5% SDS-polyacrylamide gels. Following SDS-PAGE the resolved proteins were transferred to PVDF. After blocking with the blocking solution (5% nonfat milk and 0.1% Tween 20 in TBS) for 1 h, blots were probed overnight at 4°C with gentle agitation with anti-phosphotyrosine antibody (Clone 4G10, Millipore) diluted in blocking solution at a final concentration of 0.1 μg/ml,). Afterwards, the membrane was washed thoroughly with TBS-T and then incubated for 1 h at room temperature with agitation in horseradish-peroxidase conjugated goat anti-mouse IgG diluted in blocking solution at a final concentration of 0.02 μg/ml. The membrane was then washed four times for 10 min each with TBS-T. Finally, the blot was developed using ECL detection kit according to the manufacturer’s instruction. The blot was stripped and re-probed using mouse anti-α-tubulin antibody at a final concentration of 0.02 μg/ml as internal control. Band intensity levels were measured by ImageJ software (NIH, USA). Statistical analysis was performed using the Student’s two-tailed *t*-test. In experiments where mouse anti-AKAP82-antibdy (BD Transduction Laboratories^TM^, San Jose, California) was employed, the blot was probed with the antibody at a final concentration of 0.01 μg/ml for 3 h at 4°C followed by incubation with horseradish-peroxidase conjugated goat anti-mouse IgG (0.02 μg/ml). The blot was developed using the ECL-kit.

### Immunofluorescent detection of binding of rHamOVGP1 to sperm

Aliquots of the sperm capacitated in the presence of 20 μg/ml of rHamOVGP1 for 0, 1, and 3 h, respectively, were used to examine the ability of rHamOVGP1 to bind to sperm cell surfaces. Following incubation as described above, aliquots of 20 μl of sperm were smeared onto superfrost plus microscope slides, air-dried, and fixed for 15 min in 4% paraformaldehyde. The fixed sperm samples were then labeled with a monoclonal antibody against HamOVGP1 (1 μg/ml) or with the same buffer but in the absence of the primary antibody followed by incubation in a solution of goat anti-mouse IgG-FITC at a final concentration of 2 μg/ml. After washing, the samples were mounted with 1% DABCO (1,4-diazobicyclo-[2,2,2]-octane) in 90% glycerol/PBS and then viewed on a Leica TCS-SP2 Multiphoton Confocal Laser Scanning Microscope (TCS-MP, Heidelberg, Germany).

### Immunofluorescent microscopy of capacitated sperm

Capacitated sperm (1 x 10^5^ cells) prepared as described above were smeared onto the surface of superfrost plus microscope slides, air dried, and fixed for 15 min in 4% paraformaldehyde, rinsed in PBS and then permeabilized with 0.1% Triton in PBS at 4°C overnight. In addition, samples of demembranated sperm prepared as described above were smeared onto slides, air-dried, fixed with cold (-20°C) 100% methanol for 1 min, and air-dried. Slides were then washed with DPBS containing 1% normal goat serum (NGS) and blocked with 5% NGS in DPBS for 1 h. Mouse monoclonal anti-phosphotyrosine antibody Clone 4G10 (diluted in blocking solution at a final concentration of 1 μg/ml) was applied to the slides for immunolabeling for 2 h at room temperature. Slides were then washed and incubated with goat anti-mouse IgG-FITC at a final concentration of 1 μg/ml for 1 h in the dark at room temperature. Subsequently, the slides were washed three times with DPBS containing 1% NGS and air-dried, followed by mounting with 1% DABCO in 90% glycerol/PBS. Fluorescent microscopy was performed using a Leica TCS-SP2 Multiphoton Confocal Laser Scanning Microscope (TCS-MP, Heidelberg, Germany).

### Assessment of acrosome reaction

Sperm (2 x 10^5^ cells) were incubated in TALP-PVA medium in the presence (20 μg/ml) or absence of rHamOVGP1 for different time intervals (0–6 h). Sperm were washed twice with 1ml of HBSS (5.33 mM KCl, 0.44 mM KH_2_PO4, 137.93 mM NaCl, 0.34 mM Na_2_HPO_4_, 5.56 mM D-glucose), centrifuged at 500 xg for 5 min and fixed in 100% methanol on ice for 30 min. Fixed sperm were smeared onto superfrost plus microscope slides and dried on the slide warmer. To assess the acrosomal status, samples of acrosome-reacted sperm were stained with FITC-conjugated *Pisum sativum* agglutinin (PSA) at a final concentration of 75 μg/ml. Coverslips were mounted on slides with 90% glycerol/PBS containing 1% (w/v) DABCO as an anti-bleaching agent and observed under an immunofluorescent microscope. The status of acrosome reaction was then evaluated according to the fluorescent pattern of the acrosome. Experiments were carried out in triplicates and at least 200 sperm per slide were evaluated for acrosomal status.

### Superovulation and oocyte retrieval

Four mature female hamsters weighing approximately 110 to 130g were each superovulated by intraperitoneal (i.p.) injection of 40 IU of pregnant mare’s serum gonadotropin in 100 μl saline solution (0.9% NaCl) between 9:30 and 10:00 a.m. on the day of postovualotry discharge (metestrus). Sixty hours later, the females each received an i.p. injection of 40 IU of hCG in 100 μl saline solution. Superovulated female hamsters were sacrificed 13.5 hr post-hCG injection by cervical dislocation under anesthesia after receiving an i.p. injection of sodium pentobarbital (60 mg/kg body weight). Ovaries were removed under aseptic procedures and placed in a culture dish containing TALP-HEPES-PVA. Ovarian oocytes with an expanded cumulus were collected by repeated follicle puncture using a 28-gauge needle. The oocytes with surrounding cumulus cells were transferred to TALP-HEPES-PVA medium containing hyaluronidase (1 mg/ml) and soybean trypsin inhibitor (0.01 mg/ml). Cumulus cells were removed by using a narrow-bore glass pipette, and oocytes were then washed three times in TALP-PVA medium previously equilibrated at 37°C in 5% CO_2_ in air.

### Immunolocalization of rHamOVGP1 in the ZP of ovarian oocytes

A total of 48 oocytes from mature ovarian follicles were isolated and incubated in TALP-PVA medium in the absence (24 oocytes) or presence (24 oocytes) of immunopurified rHamOVGP1 at a concentration of 20 μg/ml for 3 h at 37°C in 5% CO_2_. After incubation, oocytes were washed three times with TL-HEPES buffer, fixed for 30 min in a fixative containing 2% glutaraldehyde and 2% formaldehyde in PBS, blocked with 5% normal goat serum (NGS) in PBS for 1 h prior to incubation in primary antibody (mouse monoclonal anti-hamster OVGP1 antibody diluted in 1% NGS in PBS at a final concentration of 1 μg/ml) overnight at 4°C. Following incubation, oocytes were washed three times for 5 min each in PBS containing 1% NGS. Oocytes were then incubated in fluorescent (FITC)-conjugated goat anti-mouse IgG at a final concentration of 1μg/ml for 1 h in the dark at room temperature. Subsequently, oocytes were washed as above and air-dried, followed by mounting using 1% DABCO in 90% glycerol/PBS. Fluorescent microscopy was performed using a Leica TCS-SP2 Multiphoton Confocal Laser Scanning Microscope (TCS-MP, Heidelberg, Germany).

### Sperm-oocyte binding assay

Ovarian oocytes were incubated in 100 μl TALP-PVA with 5,000 capacitated sperm under mineral oil for 30 min. The oocytes were then washed three times with a wide-bore glass pipette to remove loosely attached sperm, and immediately fixed in a fixative containing 2% glutaraldehyde and 2% formaldehyde in PBS. Sperm bound to the oocytes were counted under the light microscope (Zeiss A x 10) at x20, and photographs were taken for documentation. In order to investigate the effect of rHamOVGP1 on sperm binding to ovarian oocytes, ovarian oocytes or sperm were preincubated with rHamOVGP1 (20 μg/ml) for 1 h (prior to sperm binding) and washed thrice with TALP-PVA medium between the preincubation and sperm-oocyte binding steps. Eleven different groups were included in this experiment as follows—Group A: capacitated sperm were incubated with oocytes for 30 min in TALP-PVA alone; Group B: pretreatment of oocytes alone with rHamOVGP1 for 1 h prior to sperm-oocyte binding; Group C: pretreatment of sperm alone with rHamOVGP1 prior to sperm-oocyte binding; Group D: rHamOVGP1 was present only during sperm-oocyte binding without prior pretreatment of oocytes or sperm; Group E: rHamOVGP1 was present during sperm-oocyte binding with pre-treated oocytes and untreated sperm; Group F: rHamOVGP1 was present during sperm-oocyte binding with pretreated sperm and untreated oocytes; Groups G to K (controls) were carried out in the same manner as Groups B to F except that monoclonal antibody against hamster OVGP1 (0.1 μg/ml) was added to the culture medium during sperm-egg binding. A total of 165 ovarian oocytes were retrieved from six animals. The 165 oocytes were randomly assigned to each of the experimental or control groups with 15 ovarian oocytes in each group.

## Results

### Production and purification of rHamOVGP1

**Fig 1:**
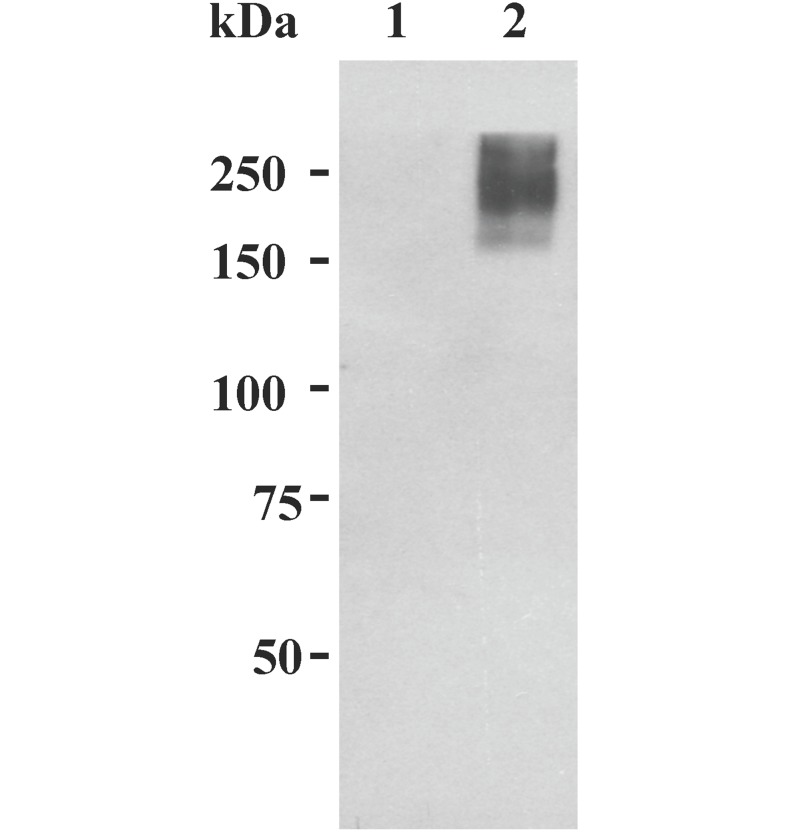
Immunochemical identification of rHamOVGP1. Culture supernatant from control (lane 1) and from rHamOVGP1-transduced HEK293 cells (lane 2) were subjected to SDS-PAGE (6.0%) followed by transfer to PVDF membranes. Western blot analysis was performed as described in Materials and Methods.

As aforementioned, oviductin (OVGP1) has been identified and characterized in several mammalian species. However, further exploration of the biological roles of OVGP1 has been hampered by the unavailability of sufficient amounts of native OVGP1 for functional and mechanistic studies. To overcome this drawback, an alternative would be to produce recombinant OVGP1 that is biologically active. It is now generally agreed that the stability and level of transgene expression from integrated lentivirus-derived vectors (LVs) are better than the traditional oncoretroviral vectors. In the present study, LV-mediated gene transfer was applied to the establishment of stable cell lines co-expressing rHamOVGP1and GFP. HEK 293 cells were infected with the transfer vector WPI-HamOVGP1. At 2-week post-infection, the individual GFP-positive clones were isolated and grown in the growth medium. The clones were screened for expression levels of rHamOVGP1 by immunoblot analysis of culture supernatants using monoclonal antibody against HamOVGP1. HEK293 cells derived from clones stably expressing high levels of rHamOVGP1 were selected and maintained in the medium and GFP expression was monitored on a daily basis. Over 90% of the cells remained GFP-positive for 12 weeks. Since rHamOVGP1 was expected to be secreted into the culture medium by HEK293 cells, we probed the presence of rHamOVGP1 in the culture medium by immunoblot analysis. The rHamOVGP1 migrated as a polydispersed band of 160–350 kDa during SDS-PAGE under reducing condition (Fig 1) consistent with results previously obtained with purified HamOVGP1 [36].

**Fig 2:**
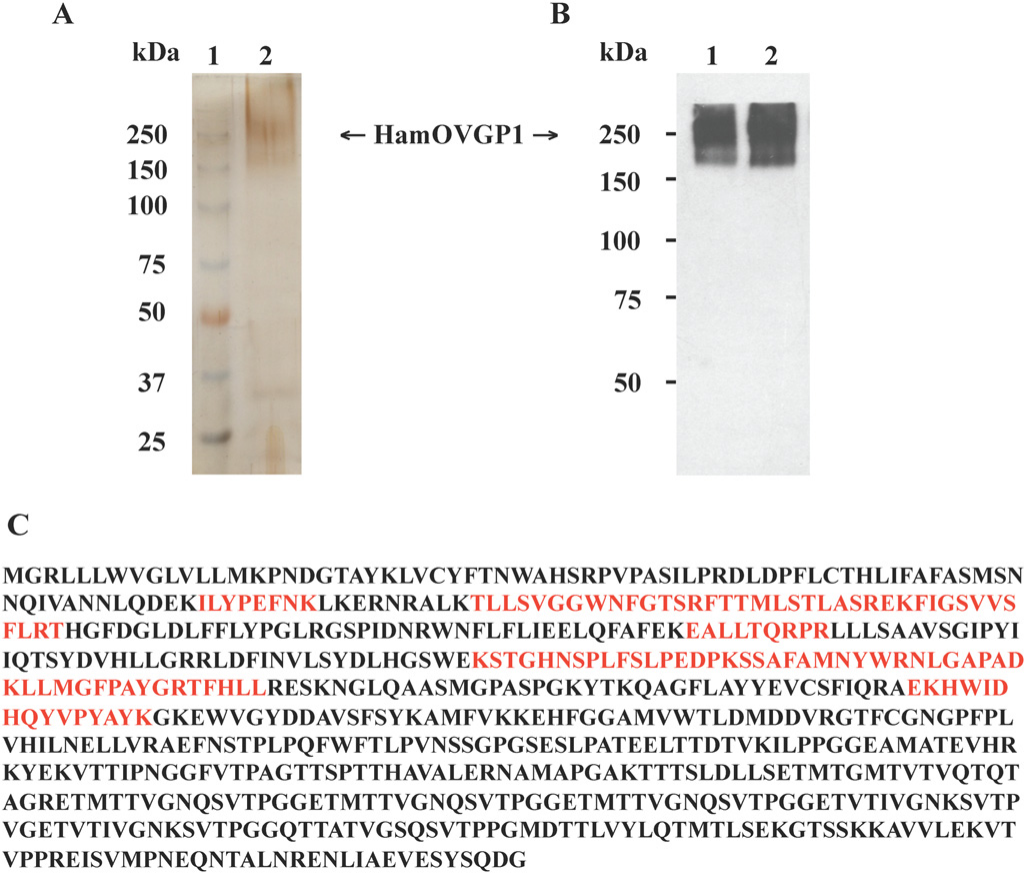
Purification and analysis of rHamOVGP1. (A) The samples were analyzed using 6% SDS-PAGE under reducing conditions followed by silver staining. Lane 1: protein ladder; lane 2: purified rHamOVGP1 (1 μg/lane) using HPA-agarose; (B) Samples were subjected to 6% SDS-PAGE followed by immunoblot analysis as described in Materials and Methods. Lane 1: purified rHamOVGP1 (100 ng/lane) using HPA-agarose; lane 2: purified HamOVGP1 (100 ng/lane) from homogenates of estrus-stage hamster oviducts using HPA-agarose; (C) Complete sequence of HamOVGP1 with the coverage of peptides (bold red) identified from the trypsin digest by MS.

At the end of the production, the culture medium was harvested and stored as described in Materials and Methods. Previous study showed that HamOVGP1 is a highly glycosylated protein that contains terminal α-D-GalNAc residues recognized by *Helix pomatia* agglutinin (HPA) [36]. This carbohydrate moiety of HamOVGP1 allowed the purification of rHamOVGP1 to homogeneity using HPA-agarose for the affinity purification of rHamOVGP1. Bound rHamOVGP1 (approx. 160–350 kDa) was eluted (Fig 2A, lane 2). Indeed, using this approach we were able to produce considerably amount of rHamOVGP1 from HEK293 cells. In our hands, a typical yield from 1L of the conditional medium varied between 0.25 to 0.5 mg of rHamOVGP1. The purity of rHamOVGP1 was assessed by SDS-PAGE (Fig 2A, lane 2) and Western blot analyses (Fig 2B, lane 1). As shown in Fig 2A, purified rHamOVGP1 revealed predominantly a single polydispersed band of the expected molecular mass of 160–350 kDa under reducing conditions (lane 2). Immunoblot analysis of HPA-purified rHamOVGP1 (Fig 2B, lane 1) was consistent with results obtained with HPA-purified HamOVGP1 from hamster oviducts prepared from the estrus stage (Fig 2B, lane 2). The identity of the purified recombinant glycoprotein was verified to be HamOVGP1 by mass spectrometric analysis (Fig 2C).

### Localization of rHamOVGP1 binding sites on hamster sperm

**Fig 3:**
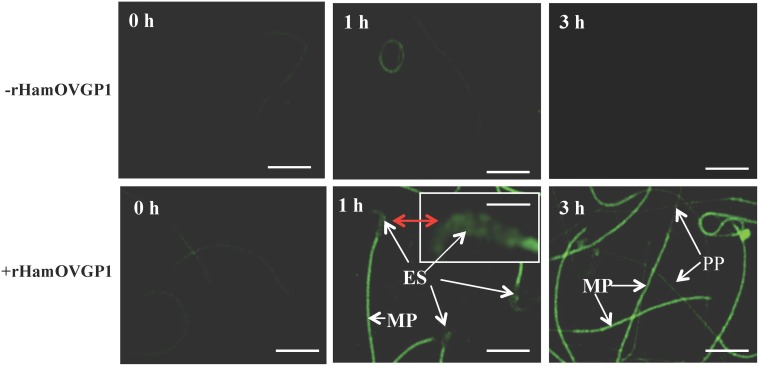
Confocal microscope imaging of rHamOVGP1 binding sites on hamster sperm after capacitation. Epididymal sperm were incubated in capacitating medium in the absence (-) or presence (+) of 20 μg/ml rHamOVGP1 for 0-, 1- and 3-h, respectively. Immunofluorescent signal was practically absent at all three time intervals in the absence of rHamOVGP1. In the presence of rHamOVGP1, immunostaining started to appear at 1 h mainly over the equatorial segment (ES) region of the sperm head (see insert for a higher magnification of the same sperm head indicated by double-head arrow in red) and the mid-piece (MP) of the sperm tail with a relatively weaker immunostaining over the principle piece (not shown). The pattern of immunostaining of the mid-piece (MP) and principle piece (PP) persisted at the 3 h time interval except that the previously seen immunofluorescent signal over the equatorial segment region now diminished or disappeared. Scale bars = 50 μm, Scale bar of insert = 140 μm

Previous immunolocalization studies carried out with OVGP1 showed the binding of HamOVGP1 to the acrosomal region of hamster sperm [28, 32] and the binding of bovine OVGP1 to the head and tail regions of bovine sperm [27]. Having successfully produced rHamOVGP1, our next step was to determine if rHamOVGP1 has the ability to bind to homologous sperm like the native HamOVGP1. Indeed, indirect immunoflurescence showed the localization of rHamOVGP1 mainly to the head and mid-piece of hamster epididymal sperm after 1 and 3 h of capacitation in a time-dependent manner (Fig 3). Immunostaining was practically absent at both time intervals when sperm were capacitated in the absence of rHamOVGP1. At 1 h, a strong immunoreaction was detected in the mid-piece of the sperm tails and in the equatorial segment region of the sperm heads (Fig 3). At 3 h, a strong immunoreaction remained associated with the mid-piece of the sperm tails with a weaker staining intensity over the principal piece. Interesting, immunostaining of the equatorial segment region previously seen at 1 h disappeared at the 3 h-time interval (Fig 3). In controls where the primary antibody was omitted, sperm samples incubated in capacitating medium even in the presence of rHamOVGP1 showed a negative immunoreaction demonstrating the specificity of the antibody (results not shown).

### rHamOVGP1 enhanced protein tyrosine phosphorylation in hamster sperm

**Fig 4:**
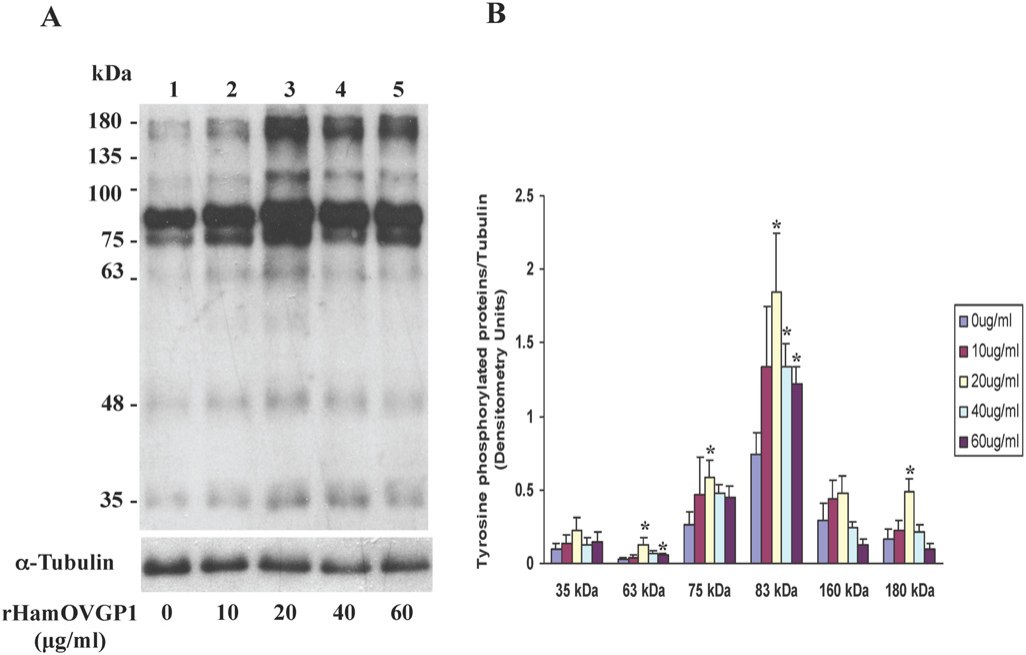
Dose-dependent effect of rHamOVGP1 on tyrosine phosphorylation of hamster sperm proteins. Hamster caudal epididymal sperm, cultured for 4 h in modified TALP medium alone or supplemented with different concentration of rHamOVGP1 (10, 20, 40, and 60 μg/ml), were processed for SDS-PAGE, transferred to PVDF membrane and probed with anti-phosphotyrosine antibody. A: representative blot; B: summary of data. Asterisks (*) indicate values that are significantly different (p<0.05) from that obtained with untreated sperm.

**Fig 5:**
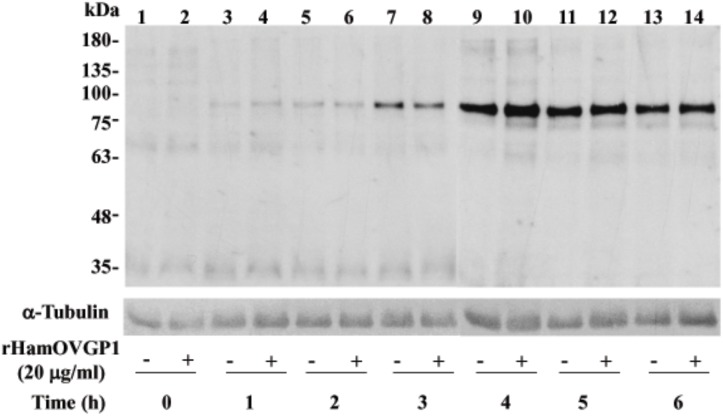
Time course of protein tyrosine phosphorylation in caudal epididymal hamster sperm. Sperm were incubated in modified TALP medium in the absence or presence of 20 μg/ml rHamOVGP1 for different time intervals (0–6 h). Equal numbers of sperm were solubilized for SDS-PAGE and immunoblotting with anti-phosphotyrosine antibody.

**Fig 6:**
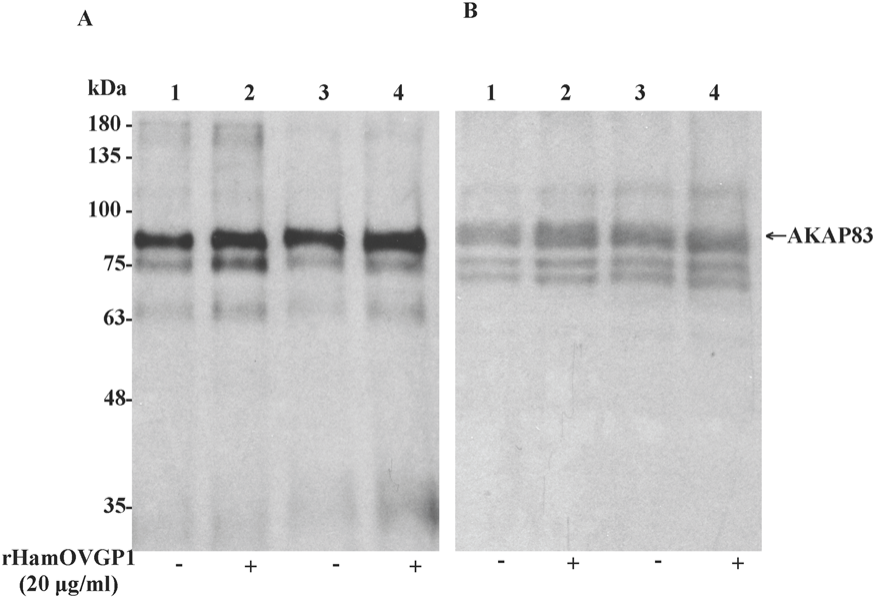
Western blot analysis of tyrosine phosphorylation of hamster sperm proteins after demembranation. (A) Hamster caudal epididymal sperm, cultured for 4 h in modified TALP medium alone (lane 1) or supplemented with 20 μg/ml of rHamOVGP1 (lane 2), were solubilized in SDS-loading buffer and subjected to immunoblot analysis with anti-phosphotyrosine antibody. Lanes 3 and 4 corresponded to lanes 1 and 2, respectively, except that sperm were demembranated with DTT-Triton X as described in Materials and Methods prior to immunoblotting. (B) The immunoblot was stripped and re-probed with anti-AKAP82 antibody as described in Materials and Methods.

A dose-dependent experiment was carried out where hamster sperm were incubated in capacitation medium (TALP-PVA) alone or supplemented with different concentrations of rHamOVGP1 (0, 10, 20, 40 and 60 μg/ml) for 4 h. Immunoblot analysis using anti-phosphotyrosine antibody indicated that the overall pattern of protein tyrosine phosphorylation in rHamOVGP1-treated and control hamster sperm was very similar (Fig 4A). But six tyrosine-phosphorylated proteins (35, 63, 75, 83, 160 and 180 kDa) exhibited an increase in labeling intensity in the treated-sperm (Fig 4A). Of the six enhanced tyrosine phosphorylated proteins the 75- and 83-kDa proteins were the most intensely enhanced phosphorylated proteins (Fig 4A). Quantification of the intensity of the major proteins showed that rHamOVGP1 at a concentration of 20 μg/ml enhanced the major tyrosine phosphorylated bands of 63, 75, 83 and 180 kDa whereas rHamOVGP1 at a concentration of 40 and 60 μg/ml only enhanced the 63kDa and 83 kDa protein bands (Fig 4B). Based on the results obtained from the dose-dependent experiment, rHamOVGP1 at a concentration of 20 μg/ml was considered the optimal concentration and was chosen for use in the time-course experiment and for the rest of the study. Our results showed that hamster sperm following incubation in TALP-PVA alone or supplemented with 20 μg/ml of rHamOVGP1 for 0–6 h exhibited a time-dependent increase in protein tyrosine phosphorylation (Fig 5). Of all the proteins, the 83 kDa protein was the most intensely phosphorylated in capacitated sperm following SDS-PAGE and immunoblot analysis (Fig 5). In this study, attempts were made to further characterize the 83 kDa protein. For this purpose, the extracts containing proteins from intact or demembranated sperm were resolved by gel electrophoresis and detected by immunoblot analysis using anti-phosphotyrosine antibody followed by stripping and re-probing using a mouse anti-AKAP82 antibody since the 83 kDa is known to be a hamster homologue of mouse AKAP82 [39]. Enrichment of sperm capacitation-associated tyrosine phosphorylated proteins by DT extraction revealed that most of them were non-membranous in nature (Fig 6A, lanes 3 and 4). A predominant 83 kDa protein was detected by anti-AKAP82 antibody (Fig 6B) corresponding to the 83 kDa protein band detected by anti-phosphotyrosine antibody (Fig 6A).

### Localization of rHamOVGP1-enhanced tyrosine phosphorylated proteins in hamster sperm

**Fig 7:**
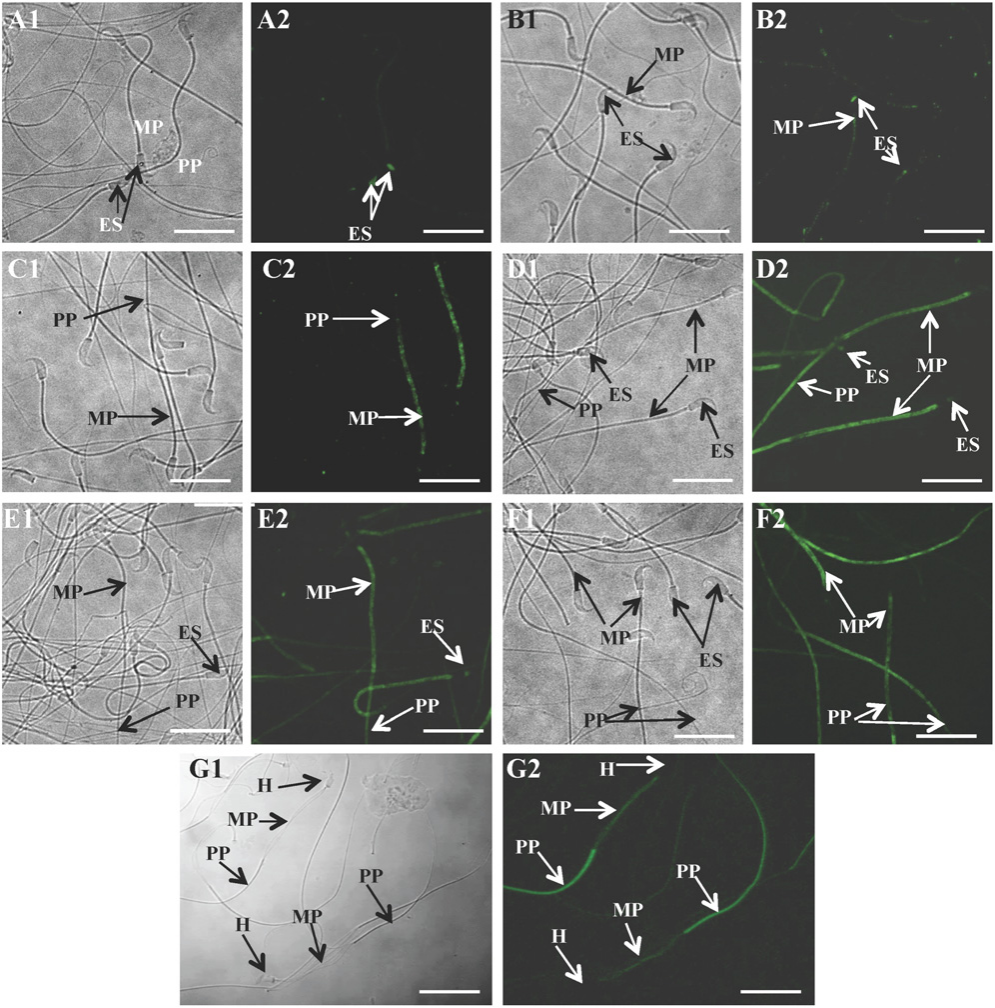
Immunolocalization of rHamOVGP1-enhanced tyrosine-phosphorylated proteins in hamster sperm. Sperm, after being cultured in modified TALP medium alone (A, C, E) or supplemented with 20 μg/ml rHamOVGP1 (B, D, F) for 0 h (A, B), 2 h (C, D) and 4 h (E, F), were subjected to indirect immunofluorescence as described in Materials and Methods. Immunofluorescent staining of tyrosine phosphorylated proteins was observed in the mid-piece (MP) and principal piece (PP). (A1-F1) are corresponding bright field photomicrographs of (A2-F2) respectively. (G) shows intense immunostaining over the principle piece (PP) with a weaker staining intensity over the mid-piece (MP) after sperm were demembranated with 2% Triton X-100 for 10 min following incubation for 4 h in modified TALP medium supplemented with 20 μg/ml rHamOVGP1. Scale bars = 50 μm

To further validate the Western blot data, sperm were subjected to immunocytochemistry using anti-phosphotyrosine antibody. Fig 7 showed a time-dependent increase of immunofluorescence signal for tyrosine phosphorylated proteins in sperm treated with 20 μg/ml of rHamOVGP1 during capacitation. Localization of tyrosine phosphorylated proteins was detected mainly in the equatorial segment region of the sperm head and along the mid-piece as well as principal piece (Figs 7B2, 7D2 and 7F2). Immunoreaction started to appear at 0 h but was localized only to the equatorial segment region of the sperm heads and not in other places (Fig 7B2). A strong intensity of immunostaining began to appear over the mid-piece with a weaker immunoreaction over the principal piece of the sperm tails at the 2 h-time interval (Fig 7D2) and this immunostaining pattern of the sperm tail persisted at 4 h of capacitation (Fig 7F2). Interestingly, immunoreaction over the equatorial segment seen at both the 0 h- and 2 h-time intervals disappeared at the 4 h-time interval (Fig 7F2). The untreated groups showed a much weaker immunofluorescence signal (Figs 7A2, 7C2 and 7E2). To substantiate the localization data, rHamOVGP1 treated-sperm were demembranated using dithiothrietol-triton which consistently removes all the membranous components leaving the cytoskeletal components of the sperm flagellum including the outer dense fibers, fibrous sheath, and axoneme intact. Demembranated sperm exhibited an intense signal in the principal piece of the sperm tail but only a faint immunofluresence signal was observed in the mid-piece (Fig 7G). These findings indicated that capacitation-associated tyrosine phosphorylated proteins are associated with membranous structures in the head region and mid-piece region, but with non-membranous components, in particular, the fibrous sheath, in the principal piece.

### Effects of rHamOVGP1 on acrosome reaction

**Fig 8:**
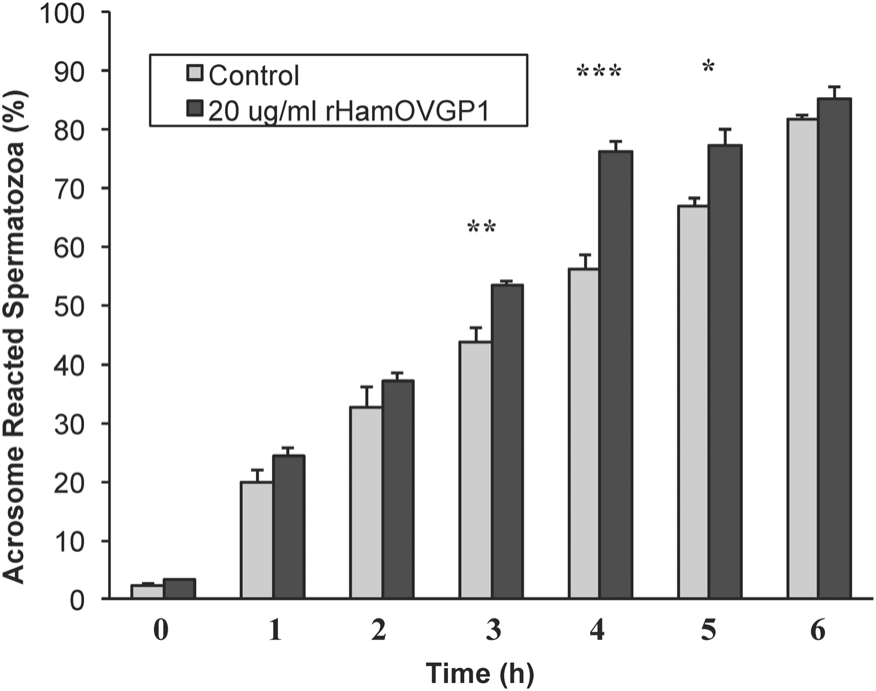
Effect of rHamOVGP1 on acrosome reaction of hamster sperm. Hamster caudal epididymal sperm were incubated in modified TALP medium alone or supplemented with 20 μg/ml rHamOVGP1 for 0 to 6 h. Results are expressed as mean ± SEM as compared to the control group (n = 4 different experiments for each group carried out under the same conditions). * p<0.05; ** p<0.01; *** p<0.001.

To find out if rHamOVGP1 has any positive effects on acrosome reaction, hamster sperm were incubated in TALP-PVA alone or in the same medium supplemented with 20 μg/ml of rHamOVGP1 for different time intervals (0–6 h). The percentage of sperm undergoing spontaneous acrosome reaction was then measured. Hamster sperm incubated in TALP-PVA alone showed a time-dependent increase in the percentage of acrosome-reacted sperm (Fig 8). A further increase in the number of acrosome-reacted sperm was noted after 3–5 h of capacitation in the presence of rHamOVGP1 compared to the controls in the absence of rHamOVGP1 (Fig 8). There was no significant difference in acrosome reaction at 6 h of capacitation since acrosome reaction of sperm incubated in TALP-PVA alone reached 80%. Therefore, rHamOVGP1 can exert a positive effect in facilitating and accelerating the process of acrosome reaction.

### Binding of rHamOVGP1 to the ZP of hamster ovarian oocytes

**Fig 9:**
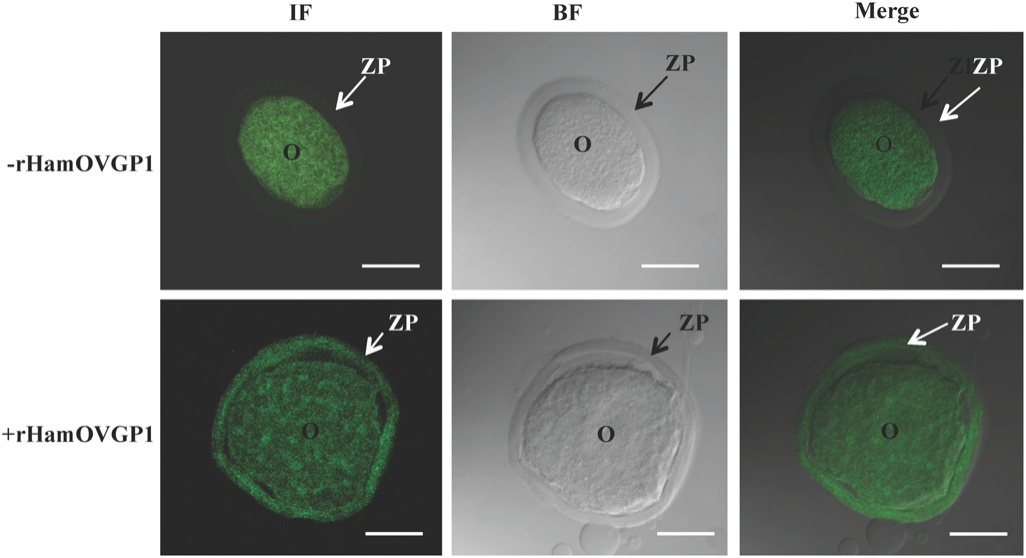
Immunolocalization of rHamOVGP1 in hamster ovarian oocytes. Confocal microscope imaging of rHamOVGP1 binding sites in hamster ovarian oocytes following 3 h of incubation in the absence or presence of 20 μg/ml rHamOVGP1. Column 1: immunofluorescent confocal microscope images; column 2: phase contrast confocal microscope images; column 3: merge of phase contrast and immunofluoresent confocal microscope images. ZP: zona pellucida; O: oocyte proper. Scale bars = 30 μm.

As expected, immunofluorescence signal was absent in the ZP of ovarian oocytes incubated in TALP-PVA medium alone (Fig 9). Bright immunofluorescence was detected throughout the entire thickness of the ZP when ovarian oocytes were incubated in culture medium containing rHamOVGP1 (Fig 9). The immunofluorescent labeling observed in the oocyte proper is considered unspecific since this was observed in both experimental and control groups.

### Influence of rHamOVGP1 on sperm binding to ZP of oocytes

**Fig 10:**
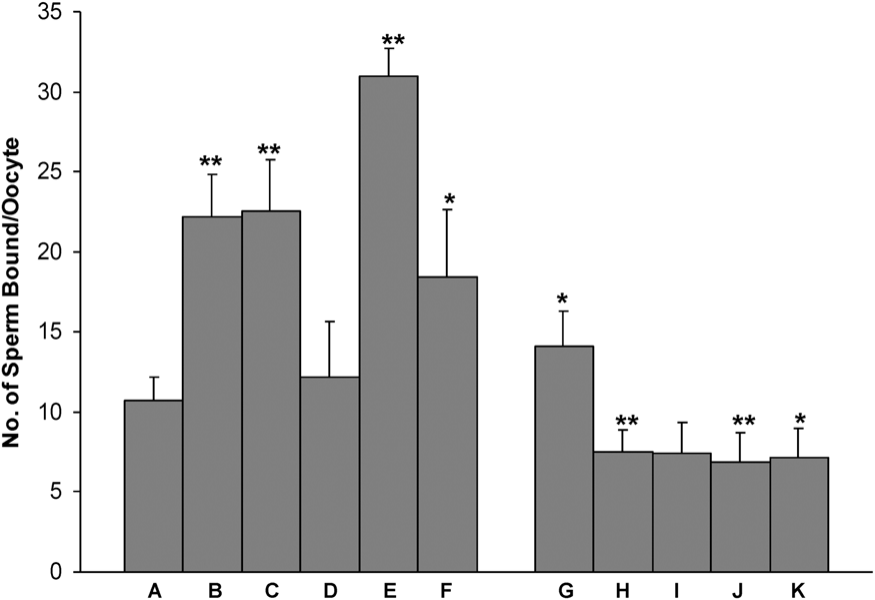
Effect of rHamOVGP1 (20 μg/ml) on sperm-oocyte binding. Group A: control (medium without additive). Group B: hamster ovarian oocytes were pretreated with rHamOVGP1 for 1 h prior to sperm-oocyte binding assay in the absence of rHamOVGP1. Group C: hamster caudal epididymal sperm were pretreated with rHamOVGP1 for 1 h prior to sperm-oocyte binding assay in the absence of rHamOVGP1. Group D: Sperm-oocyte binding assay was performed in the presence of rHamOVGP1 without prior pretreatment of either oocytes or sperm. Group E: Sperm-oocyte binding assay was performed in the presence of rHamOVGP1 with pretreated oocytes and untreated sperm. F: Sperm-oocyte binding assay was performed in the presence of rHamOVGP1 with pretreated oocytes and untreated oocytes. Groups G to K correspond to Group B to F, respectively, except that monoclonal antibody against hamster OVGP1 was added to the culture medium during sperm-oocyte binding. Data are presented as Mean ± SEM of the number of sperm bound per oocyte. A total of 165 oocytes were obtained from six animals. Fifteen ovarian oocytes were present in each group. Group B to F are compared to Group A; Group G to K are compared to Group B to F, respectively. * p<0.05; ** p<0.001.

In order to determine if rHamOVGP1 affects sperm-oocyte binding, pre-treatment of sperm and/or oocytes with rHamOVGP1 were carried prior to incubation in TALP-PVA medium in the presence or absence of rHamOVGP1. The mean number of hamster sperm bound to hamster ovarian oocytes in TALP-PVA medium alone was 10 ± 1 sperm/oocyte (Fig 10: Group A). A significant increase in the number of sperm bound to the oocytes was found when either oocytes (Fig 10: Group B) or sperm (Fig 10: Group C) were pre-treated with rHamOVGP1 prior to incubation in TALP-PVA alone (22 ± 2 and 22 ± 3 sperm/oocytes, respectively, with p<0.05). Interestingly, when both previously untreated oocytes and sperm were incubated in TALP-PVA in the presence of rHamOVGP1 (Fig 10: Group D), the number of sperm bound per oocytes (12 ± 2) was comparable to that of the control (10 ± 1) with no significant increase. A significant increase was noted when either pre-treated oocytes (Fig 10: Group E) or sperm (Fig 10: Group F) were incubated in the medium in the presence of rHamOVGP1 albeit with a larger increase in the pre-treated oocyte group (31 ± 3, p<0.01 vs. 18 ± 4, p<0.05). Addition of monoclonal antibody against hamster OVGP1 to the medium during sperm-oocyte binding incubation in experimental groups B to F abolished the enhancement of sperm binding to oocyte by rHamOVGP1 (Fig 10: Groups G to K). In addition, we also performed an experiment in our study where both sperm and oocytes were pre-treated with rHamOVGP1 prior to sperm-oocyte binding assay. However, the increase was found to be not statistically significant as compared to the control (result not shown).

## Discussion

Mammalian oviductal fluid consists of a complex mixture originating from plasma transudation and glycoproteins secreted by oviduct epithelial cells [40]. Accumulated evidence has shown that OVGP1, also known as oviduct-specific glycoprotein or oviductin, a major glycoprotein secreted by the oviductal epithelium, plays important roles during the early events of fertilization and early embryo development [24]. Although OVGP1 has been identified and characterized in many mammalian species including the human, further elucidation of its biological functions and the study of the mechanism regulating its functions have been hampered by the limited quantities of OVGP1 that can be purified from oviductal fluid and/or oviductal cells.

In the present study, we used recombinant technology to successfully produce rHamOVGP1. It has been previously shown that HamOVGP1 contained terminal α-D-GalNAc residues [6] and can be purified using HPA-agarose affinity columns [36]. Using the similar approach, we purified from HEK293 culture medium the rHamOVGP1 that displayed a polydispersed band of 160–350 kDa on SDS-PAGE gel, which is consistent with the molecular mass of purified native protein previously described [36]. The identity of the recombinant glycoprotein was further confirmed by immunoblot analysis using antibody generated against HamOVGP1 and subsequently by mass spectrometric analysis.

The main goal of producing rHamOVGP1 is to obtain sufficient quantities of the recombinant glycoprotein for functional and mechanistic studies, which, otherwise, cannot be attained with limited amount of native HamOVGP1. Therefore, having successfully produced rHamOVGP1 it would be imperative to determine if the newly generated rHamOVGP1 is biologically active. First, we set out with an experiment to determine if rHamOVGP1 has the ability to bind to the sperm surfaces since previous studies showed the binding of native hamster [41] and bovine [42] OVGP1, respectively, to the head regions of corresponding homologous sperm. Indeed, our results with indirect immunofluorescence showed localization of rHamOVGP1 mainly to the sperm head and to the middle piece of the sperm tail with a lesser extent to the principal piece. The present results suggest that rHamOVGP1 may exert its function through its binding to both the head and tail regions of hamster sperm. This notion is particularly appealing since a recent study carried out in our laboratory showed that native HamOVGP1 purified from hamster oviducts can enhance sperm capacitation by potentiating tyrosine phosphorylation of several sperm proteins known to be involved in sperm-egg binding and sperm motility that are associated with the sperm head and tail, respectively. [32]. In the present rHamOVGP1 binding experiment, it was also observed that the immunostaining of the head region previously seen at 1 h greatly diminished or disappeared at the 3 h-time interval. This could be attributed to the high percentage of sperm undergoing spontaneous acrosome reaction in the hamster species as previously reported [43]. As shown in Fig 8, 45 to 55 percent of hamster sperm were found to be acrosome-reacted between the 3 h- and 4-h time intervals. Therefore, the reduction or disappearance of the immunostaining at the 3 h-time interval was likely due to the acrosome-reacted status of the sperm heads.

In order to become fertilizing competent, mammalian sperm freshly coming from the epididymis must undergo a process called “capacitation” which occurs either normally in the female reproductive tract or *in vitro* under an appropriate environment [44–47]. Capacitation is characterized by sperm exhibiting hyperactivated motility, and is associated with several molecular changes including increases in intracellular Ca^2+^, HCO_3_^-^, and cAMP levels, an efflux of cholesterol, and changes in protein kinase and phosphatase activities [48, 49]. Among these physiological changes characteristic of sperm capacitation, an increase in global tyrosine phosphorylation (PYP) of sperm proteins has been shown to be a hallmark of capacitation [50, 51]. Mammalian fertilization resulting from fusion of an oocyte with a capacitated sperm represents the standard endpoint of capacitation. Accumulated evidence points to the fact that acquisition of hyperactivation by sperm during capacitation, an event necessary for fertilization, is under the influence of OVGP1[40]. We have recently demonstrated, for the first time, that native estrus-associated HamOVGP1 can enhance sperm capacitation by increasing tyrosine phosphorylation of a subset of sperm proteins in a time-dependent manner [32]. Therefore, in the present study an experiment was carried out to determine if rHamOVGP1 has the ability to enhance sperm capacitation by increasing tyrosine phosphorylation of sperm proteins similar to what we previously observed with the native HamOVGP1 [32]. As expected, our results demonstrated that the rHamOVGP1 that we produced is biologically potent and capable of enhancing tyrosine phosphorylation of sperm proteins like the native HamOVGP1. Particularly rHamOVGP1 at a concentration of 20 μg/ml appears to be the optimal concentration which significantly enhanced several proteins of 63, 75, 83 and 180 kDa in a time-dependent manner with a peak at 4 h of capacitation (Figs 5 and 6). The present results demonstrate clearly that the effect of rHamOVGP1 on protein tyrosine phosphorylation (PYP) during capacitation is dose- and time-dependent.

Having obtained the results above, it would be of interest to identify the enhanced tyrosine phosphorylated proteins that are under the influence of rHamOVGP1. Of all the tyrosine phosphorylated proteins enhanced by rHamOVGP1, the 83 kDa protein was found to be the most intensely phosphorylated reaching a peak at 4 h of capacitation. This protein was subsequently revealed by immunoblot analysis to be a kinase anchoring protein (AKAP83, also called AKAP4), a major component of the fibrous sheath previously shown to be a major tyrosine phosphorylated protein in capacitated hamster sperm [39]. Importantly, we have now reported, for the first time, that this protein (AKAP83) is further enhanced by rHamOVGP1 during capacitation substantiating the involvement of AKAP83 in capacitation and its possible role in sperm motility due to its localization in the principle piece of the sperm tail. In our study, immunofluorescent staining of demembranated sperm localized the rHamOVGP1-enhanced, tyrosine phosphorylated proteins exclusively to the principal piece of hamster sperm flagella. Therefore, the present immuno-localization results confirm the identity of the rHamOVGP1-enhanced 83 kDa protein as a fibrous sheath protein. Prior experimental evidence suggests that the fibrous sheath not only acts as a passive elastic structural element of the sperm tail but may also be an active regulator of flagellar movement essential for sperm motility [52]. The authors of the study postulated that tyrosine phosphorylation of fibrous sheath components of the sperm tail could lead to a decrease in the stiffness of fibrous sheath and thus enabling the flagellum to generate greater bends leading to sperm hyperactivation. To date, despite extensive study of mammalian sperm motility, the mechanism underlying sperm hyperactivation is still not fully understood. Our present experimental results revealing the 83 kDa protein as the AKAP83 fibrous sheath protein provide further, albeit indirect, support for a role of tyrosine phosphorylation of sperm proteins in sperm hyperactivation. More importantly, the present results showing the rHamOVGP1-enhanced tyrosine phosphorylation level of several major proteins, in particular the 83 kDa protein, reinforces the notion that the oviductal lumen, where OVGP1 is present *in vivo*, provides a suitable milieu for sperm to prepare their way for fertilization.

Since capacitation prepares sperm to undergo acrosome reaction, a study was undertaken to determine if prior capacitation of hamster sperm in the presence rHamOVGP1 can increase the number of acrosome-reacted sperm as compared to their counterpart capacitated in the absence of rHamOVGP1. The spontaneous acrosome reaction in active motile sperm can be used as an indication of the completion of capacitation in hamster sperm. Indeed, using this parameter the present results showed a significant and time-dependent increase in the number of acrosome-reacted sperm when sperm were capacitated in TALP-PVA medium in the presence 20 μg/ml of rHamOVGP1 at 3 h, 4h and 5 h time intervals.

Previous studies using oviductal fluid or partially purified OVGP1 from various species, including the hamster, showed the binding of OVGP1 to the ZP of corresponding post-ovulatory oocytes [11, 24, 25, 36]. Endogenous form of HamOVGP1 has been found to associate with the ZP of ovulated oocytes once it is released from the oviductal epithelial cells [25]. Oocytes isolated from mature ovarian follicles incubated with immunopurified endogenous HamOVGP1 displayed a strong immunofluorescent signal localized in the ZP of hamster oocytes [28, 36]. Therefore, if the rHamOVGP1 that we produced is biologically active, it should also possess the ZP-binding property. Indeed, results of our immunostaining experiments showed that rHamOVGP1 behaved like its endogenous counterpart and bound uniformly throughout the entire thickness of the ZP of hamster ovarian oocytes that had not been previously exposed to the oviductal environment (Fig 9). The immunofluorescent signal observed in the oocyte proper was likely due to nonspecific binding of *the* secondary antibody to the oocyte proper since this signal was observed regardless of the presence or absence of rHamOVGP1 and the monoclonal anti-HamOVGP1 antibody.

Having demonstrated that rHamOVGP1 is able to bind the ZP of ovarian oocytes, we hypothesized that rHamOVGP1 can also increase sperm-oocyte binding. In the present study, pre-treatment of either oocytes or sperm with rHamOVGP1 prior to incubation in TALP-PVA alone significantly increased the number of sperm bound per oocyte. This increase was also observed when rHamOVGP1 was present during both oocyte or sperm pre-incubation and sperm-oocyte binding. The addition of rHamOVGP1 in TALP-PVA medium without pre-treatment of oocytes and sperm, however, did not seem to affect sperm-oocyte binding. Therefore, the positive effect of rHamOVGP1 on sperm-oocyte binding appears to be more associated with the oocyte since the largest increase in the number of sperm bound to oocytes was noted when the oocytes were pre-exposed to rHamOVGP1. The addition of monoclonal HamOVGP1 antibody in the medium negated the positive effect of rHamOVGP1 on sperm-oocyte binding thus confirming the specificity of rHamOVGP1. The present results are in agreement with the overall concept that the effects of mammalian OVGP1 on reproductive events are mediated primarily through interactions with the oocyte [24]). It is noteworthy to mention OVGP1-null mice have been reported to be fertile *in vivo* [53]. However, protein expression of mouse OVGP1 was not carried out in that study [53]. Furthermore, information on mouse OVGP1 is relatively limited compared to that found in other mammals including the human, particularly at the protein level. Contrary to other mammals where OVGP1 is detected in the ZP of postovulatory oocytes, an oviduct-derived glycoprotein reactive with wheat germ agglutinin was found in the perivitelline space but not in the ZP of mouse oocytes and early embryos [54]. Although a latter study using antibody against human OVGP1 localized the corresponding antigen to both the ZP and perivitelline space of mouse oocytes [55], the identity and biological role of OVGP1 in the mouse species remain unclear.

Importantly, in the present study our results directly link the increase in tyrosine phosphorylation of sperm proteins to the beneficial effect of OVGP1 on sperm capacitation. Although capacitation of mammalian sperm, including human sperm, can be achieved *in vitro* under appropriate conditions, an exhaustive search on major suppliers of commercially available capacitating media reveals that commercially available capacitating media do not contain OVGP1. Capacitation is a fundamental process that normally occurs in the lumen of the female reproductive tract where OVGP1 is present. Our data showing that pre-treatment of sperm and/or oocytes with OVGP1 can enhance sperm capacitation through stimulation of tyrosine phosphorylation of sperm proteins and increase sperm-oocyte binding are novel and important findings that reinforce the biological significance of OVGP1 *in vitro*. It is envisaged that future development and refinement of recombinant OVGP1 in other mammalian species, including the human, will allow us to improve and optimize culture conditions for early embryo development and *in vitro* fertilization procedures.

## Conclusions

In summary, results obtained with our newly produced rHamOVGP1 showed that addition of rHamOVGP1 in the capacitation medium further enhanced tyrosine phosphorylation of a sub-set of sperm proteins and significantly increased acrosome reaction. Interestingly, one of the most enhanced tyrosine phosphorylated protein during sperm capacitation under the influence of rHamOVGP1 is AKAP83, a major fibrous sheath protein. Furthermore, results obtained in the present study validated our hypothesis that rHamOVGP1 is biologically active and that pre-treatment of oocytes and sperm with rHamOVGP1 can increase sperm-oocyte binding. The successful large-scale production of rHamOVGP1 and the positive effects of rHamOVGP1 on sperm functions and sperm-oocyte binding will pave way for further identification and characterization of capacitation-associated and rHamOVGP1-enhanced tyrosine phosphorylated proteins and for further elucidation of its role in the early events of fertilization. Future studies are also warranted to investigate the mechanisms that regulate the physiological function of rHamOVGP1, for instance, the role of glycan derivatives of HamOVGP1 on early events of fertilization.
